# Transcriptome analysis reveals the molecular mechanisms of rubber biosynthesis and laticifer differentiation during rubber seed germination

**DOI:** 10.3389/fpls.2024.1337451

**Published:** 2024-01-24

**Authors:** Bin Hu, Na Yang, Zaihui Zhou, Xiangyu Shi, Yunxia Qin, Yongjun Fang, Xiangyu Long

**Affiliations:** ^1^ National Key Laboratory for Tropical Crop Breeding, Ministry of Agriculture Key Laboratory of Biology and Genetic Resources of Rubber Tree, State Key Laboratory Breeding Base of Cultivation and Physiology for Tropical Crops, Rubber Research Institute, Sanya Research Institute of Chinese Academy of Tropical Agricultural Sciences, Haikou, China; ^2^ National Key Laboratory of Crop Genetic Improvement and National Center of Plant Gene Research (Wuhan), Huazhong Agricultural University, Wuhan, China; ^3^ Danxin College, Hainan University, Danzhou, China

**Keywords:** rubber synthesis, laticifer differentiation, seed germination, transcriptome, transcription factors

## Abstract

The molecular mechanisms underlying the initiation of natural rubber synthesis and laticifer differentiation have not been fully elucidated. In this study, we conducted a time-series transcriptome analysis of five rubber tree tissues at four stages of seed germination. A total of 161,199 DEGs were identified between the two groups, including most 16,673 DEGs (A3 vs B3 and A3 vs C3) and lest 1,210 DEGs (C2 vs D2). We found that the maturation of the seed is accompanied by the formation of laticifer cells in cotyledon. Meanwhile, the analysis of hormones related genes expression may provide effective clues for us to promote the differentiation of laticifer cells in seeds by hormones in the future. In this study, hormone-related gene enrichment analyses revealed that IAA, GA, and CTK were activated in laticifer containing tissues. Similarly, GO and GEGG analysis showed that hormone pathways, especially the auxin pathway, are enriched. Gene expression clustering was analyzed using the short time-series expression miner (STEM), and the analysis revealed four distinct trends in the gene expression profiles. Moreover, we enriched transcription factor (TF) enrichment in cotyledon and embryonic axis tissues, and the MYB type exhibited the most significant difference. Furthermore, our findings revealed that genes related to rubber synthesis exhibited tissue-specific expression patterns during seed germination. Notably, key genes associated with rubber biosynthesis, specifically *small rubber particle protein* (*SRPP*) and *cis-prenyltransferase* (*CPT*), exhibited significant changes in expression in cotyledon and embryonic axis tissues, suggesting synchronous rubber synthesis with seed germination. Our staining results reveled that laticifer cells were exits in the cotyledon before seed imbibition stage. In conclusion, these results lay the foundation for exploring the molecular mechanisms underlying laticifer differentiation and rubber synthesis during seed germination, deepening our understanding of the initiation stages of rubber biosynthesis and laticifer differentiation.

## 1 Introduction

There are more than 2,500 species of rubber-producing plants worldwide. The rubber tree (*Hevea brasiliensis*) is a globally significant economic tree crop, and its latex is utilized in diverse fields, such as medicine, transportation, and high-tech research (Herculano et al., 2009; Guerra et al., 2021). Latex is typically obtained by tapping the trunks of mature rubber trees, with laticifers responsible for rubber production found in the phloem (d'Auzac, 2018).

Rubber is a polyisoprene macromolecule synthesized via the mevalonate pathway (MVA) rather than the phosphoenolpyruvate (PEP) pathway in laticifers (Sando, T., et al., 2008a). In laticifer cells, IPP is polymerized by CPT to form natural rubber (Cherian et al., 2019; Yamashita and Takahashi, 2020; Calvo-Polanco et al., 2021). Latex is a mixture of polyisoprene macromolecules that are synthesized in specialized laticifer cells. Previous studies have indicated relatively high expression of MVA pathway-related genes in rubber latex compared to other tissues (Sando, T., et al., 2008b). Additionally, key genes involved in rubber synthesis, such as *CTP*, *CBP*, and *SRPP*, exhibit tissue-specific expression in latex (Long et al., 2021). Recent research findings suggest that heterogeneous *CPTs* can promote natural rubber (NR) elongation in the endogenous *CPT*-deficient cellular context of lettuce laticifers (Kwon et al., 2023). Similarly, *SRPP1* overexpression affects chain elongation in *E*. *ulmoides* Oliver, impacting particle development and rubber content (Ran et al., 2022). Sugar metabolism provides precursors for rubber synthesis, and *HbNIN2* (a cytosolic alkaline/neutral invertase), *HbSUT5* (a sucrose transporter), and *HbHXK2* influence rubber synthesis via the sugar metabolism pathway (Liu et al., 2015; Long et al., 2019; Fang et al., 2021). As the primary site of latex synthesis, the number of laticifers plays a critical role in rubber production. Recent research has shown that *HbMYC26* and *HbPSK5* (phytosulfokines) are positive regulatory factors of laticifer numbers (Chao et al., 2023), suggesting that *HbMYC26* and *HbPSK5* may be involved in laticifer differentiation.

Phytohormones play crucial roles in plant regulation, including cell differentiation, tissue development, and the response to various stresses (Cackett et al., 2022; Waadt et al., 2022). There are few studies on the role of hormones in the rubber biology of rubber trees; however, some hormones participate in rubber production. Ethephon effectively increases rubber production, while abscisic acid (ABA), salicylic acid (SA), and auxins serve as efficient yield stimulants in mature untapped rubber trees (Tungngoen et al., 2011; Liu, 2016). Moreover, several genes are involved in laticifer cell differentiation. IAA promoted laticifer differentiation *in vitro* (Suri and Ramawat, 1995). Previously, scientists thought JA promoted the differentiation of laticifer cells, but a recent study suggested that JA stimulated latex production but had no effect on laticifer differentiation (Castelblanque et al., 2018). Therefore, the effects of plant hormones on latex production and laticifer differentiation need further study.

Rubber-producing plants include two types of laticifers: articulated and non-articulated (Hagel, Jillian M. et al., 2008). Non-articulated laticifers originate from single cells or primordia and can be found in immature embryos. In contrast, articulated laticifers develop from multiple connected cells, as observed in mature rubber tree laticifers. Prior studies have focused primarily on laticifer development during the seedling or maturity stages, but the understanding of laticifer initiation has been limited. Furthermore, the expression of rubber synthesis-related genes in different tissues of the rubber tree during various seed germination stages has not been determined. In this study, we compared transcriptomic profiles during seed germination at four stages—seed imbibition, seed coat rupture, radicle protrusion, and seedling emergence—across five tissues: endosperm, cotyledon, embryonic axis, radicle, and plumule. This study presents the first transcriptome dataset for rubber seed germination, providing insights into the initiation of rubber synthesis and laticifer differentiation at the transcriptional level during seed germination.

## 2 Materials and methods

### 2.1 Plant materials and sample collection

For this study, we utilized seeds from the rubber tree variety GT-1 (Gondang Tapen 1), which is known for its excellent yield traits. Seeds were obtained from the experimental farm of the Chinese Academy of Tropical Agricultural Sciences (Danzhou, Hainan, China) and rubber seeds soaked in water until seed coat rupture stage, and then rubber seed of radicle protrusion and seedling emergence stages planted in sandy soils within a controlled chamber at the Rubber Research Institute, Chinese Academy of Tropical Agricultural Sciences, Haikou, China. The sandy soil was kept consistently moist, and the ambient temperature was maintained at 20°C (Yan and Cao, 2009). We collected samples from five tissues—the endosperm, cotyledon, embryonic axis, radicle, and plumule—during four stages of seed germination: seed imbibition, seed coat rupture, radicle protrusion, and seedling emergence. Three biological replicates were performed for each tissue.

### 2.2 RNA extraction

Total RNA was promptly extracted on the same day as the samples were collected and stored at –80°C for further analysis. The rubber seeds of radicle protrusion and seedling emergence stage planted in sandy soil, and the tissue samples such as endosperm, cotyledon, embryonic axis, radicle or plumule separated from the seeds that take out from sandy soil and be cleaned, and flash-frozen in liquid nitrogen immediately after collection. Subsequently, the samples were placed in a mortar within liquid nitrogen and then grinded to powder in liquid nitrogen, and total RNA was extracted using TRIzol reagent (TransGen Biotech, Beijing, China) following the manufacturer’s instructions (Tang et al., 2010).

### 2.3 Transcriptome sequencing and data analysis

RNA extracted from various tissue samples at different germination stages was subjected to transcriptome sequencing. For the total RNA, three biological replicates were pooled. Transcriptome sequencing was conducted by Majorbio Technology Corporation using the NovaSeq 6000 system following the Illumina Sequencing Protocol. The quality of the raw reads was checked using ‘FastP’ (Chen et al., 2018). High-quality reads from each sample were aligned to the rubber tree reference genome available at https://ftp.ncbi.nlm.nih.gov/genomes/all/GCF/001/654/055 (Kim et al., 2015) using ‘HISAT2’. The aligned reads were subsequently utilized to construct a reference annotation-based transcript assembly, enabling the identification of uniquely mapped reads for each sample. The results were subjected to ‘featureCounts v1.5.0’ (Liao et al., 2014) to obtain read counts for all samples. Differences in gene expression were assessed using DESeq2 1.2.5R (Love et al., 2014), with the p-value and false discovery rate (FDR) adjusted p-value (or q-value) set at 0.05. Gene Ontology (GO) enrichment analyses, including the categories of biological process, cellular component, and molecular function, as well as Kyoto Encyclopedia of Genes and Genomes (KEGG) pathway enrichment analyses, were performed on the differentially expressed genes (DEGs) across the samples using the clusterProfiler R package (Yu et al., 2012).

### 2.4 Identification of differentially expressed genes

The expression levels of each transcript across the 44 samples were calculated and normalized to fragments per kilobase per million mapped reads (FPKM). DESeq2 was used to identify DEGs according to the following criteria: FDR < 0.05 and fold change > 2. Subsequent enrichment analyses included KEGG and GO enrichment analyses, as well as reference to the Plant Transcription Factor Database (PlantTFDB 4.0) http://planttfdb.cbi.pku.edu.cn/.

### 2.5 Observation of laticifers cells in different tissues

Fresh samples of endosperm, embryonic axis, radicle and plumule from seeds of seedling emergence stage and cotyledon from freshly harvested seeds were fixed in 80% (v/v) ethanol for 24 h and then sliced by a vibratome (Leica, Germany). The vibratome parameters were as follows: speed = 1.00mm/s, amplitude =1.0mm, and thickness = 100 μm. Sections were stained with Oil Red O (Sigma, USA) and mounted in 60% (v/v) glycerol (Lin et al., 2022). Pictures were taken with a light microscope SZX16 (Olympus, Japan).

### 2.6 Validation of candidate genes by real-time quantitative PCR

Total RNA was reverse-transcribed into cDNA using the PrimeScript 1st Strand cDNA Synthesis Kit (Takara, Dalian, China). RT−qPCR amplification was carried out using SYBR^®^ Premix Ex Taq™ II (Perfect Real Time) (Takara, Dalian, China) and the CFX96 Touch™ Real-Time PCR Detection System (Bio-Rad, Hercules, CA, USA) following the manufacturer’s protocol. The mitosis protein YLS8 (Long et al., 2016) served as an endogenous control for the normalization of RT−qPCR Ct values, and relative gene expression levels were determined using the ΔΔCt method (Livak et al., 2001). Gene-specific primers were designed utilizing the NCBI Primer-BLAST tool (http://www.ncbi.nlm.nih.gov/tools/primer-blast/). The primer sequences for the three candidate genes are listed in **Supplementary Table 1**.

### 2.7 Statistical analysis

The results are presented as the mean ± standard error (SE), and three technical replicates used in qPCR, and the statistical analyses were performed using SPSS v17.

## 3 Results

### 3.1 Sample acquisition and high-throughput total RNA sequencing

A total of 44 RNA-Seq libraries were constructed for five tissues of the rubber tree (**Supplementary Figure 1A**), including the endosperm, cotyledon, embryonic axis, radicle, and plumule, at four different germination stages (**Supplementary Figure 1B**). We obtained 306.32 Gb of clean data from the 44 libraries representing five tissues: 12 endosperm samples, 11 cotyledon samples, 12 embryonic axis samples, 6 radicle samples, and 3 plumule samples at 4 stages. The clean data from each sample exceeded 5.95 Gb, with more than 92.53% of the reads being Q30 bases. More than 92.22% of the clean reads were successfully mapped to the rubber tree reference genome (*Hevea brasiliensis*).

### 3.2 GO and KEGG enrichment analyses of differentially expressed genes

Differentially expressed genes (DEGs) were identified in the endosperm, cotyledon, and embryonic axis samples at various stages of seed germination. The number of DEGs ranged from 255 (C1_vs_D1) to 16,673 (A3_vs_B3) (**Supplementary Table 2**). Across all four stages, 41 genes were expressed in the cotyledonous tissue (**Supplementary Figure 2B**), 24 genes were expressed in the embryonic axis (**Supplementary Figure 2C**), and no genes were enriched in the endosperm (**Supplementary Figure 2A**). Similarly, compared with the other groups, the A1 vs. B1, A1 vs. C1, and A1 vs. D1 groups shared the largest number of DEGs (3451). This trend was also observed in the A2 vs. B2, A2 vs. C2, and A2 vs. D2 groups, which included 7875 DEGs, as well as in the A3 vs. B3, A3 vs. C3, and A3 vs. D3 groups, which included 8686 DEGs. These results indicated that most of the genes were expressed during the early stages of seed germination compared to the later three stages.

**Figure 1:**
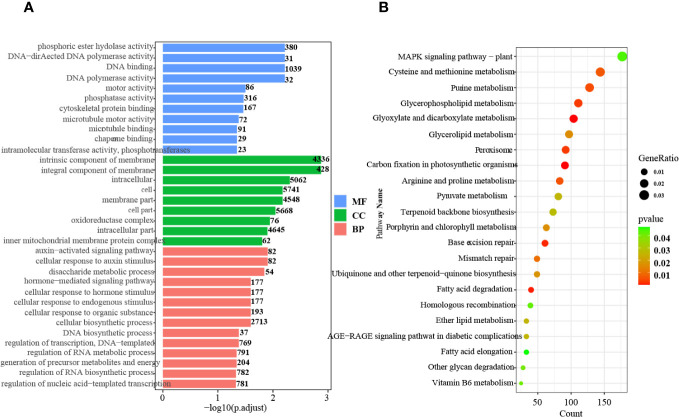
GO and KEGG pathway enrichment analysis of the DEGs. **(A)** GO enrichment analysis of up- and downregulated genes in all tissues and stages. MF, Molecular function; CC, Cellular component; BP, Biological process. **(B)** KEGG pathway enrichment analysis of up- and downregulated genes in all tissues and at all stages.

GO and KEGG pathway enrichment analyses of the DEGs were also conducted. A total of 34 highly significant terms were identified, including 11 for molecular function, 9 for cellular component, and 14 for biological process (**Figure 1A**).

In the biological process category, pathways related to auxin signaling, cellular response to auxin stimulus, and hormone-mediated signaling were significantly enriched. This finding indicates the critical involvement of hormones, particularly auxin, throughout all stages of seed germination. Furthermore, KEGG pathway analysis demonstrated enrichment in 22 pathways, including terpenoid backbone biosynthesis, ubiquinone and other terpenoid-quinone biosynthesis, peroxisome, and MAPK signaling (**Figure 1B**). These results indicate that active terpenoid metabolism occurs during seed germination and potentially serves as the foundation for rubber synthesis.

### 3.3 DEGs associated with phytohormone signaling pathways

Phytohormones, including auxin, abscisic acid (ABA), gibberellin (GA), and cytokinin (CTK), are critical regulators of seed germination and crop development (Liu et al., 2007; Miransari and Smith, 2014; Oracz and Karpinski, 2016; Xue et al., 2021; Yang et al., 2022). They also have the potential to influence laticifer development. Therefore, we obtained phytohormone-related genes http://hevea.catas.cn/home/index, specifically auxin, ABA, GA, and CTK, from in different tissues and at four stages of seed germination to analyze the relationship between laticifer differentiation and plant hormones.

#### 3.3.1 Auxin-related genes strongly activated at later three stages

Auxin, a crucial phytohormone in embryonic development (Liu et al., 2013; Matilla, 2020), exhibited minimal expression during the seed imbibition stage but became more prominent as seed germination progressed, especially during the radicle protrusion and seedling emergence stages. Some genes were exclusively expressed during the seed imbibition stage, including six auxin-responsive genes (e.g., *scaffold2057_27488*, *scaffold0628_462345*, *scaffold0563_748886*, *scaffold0375_663805*, *scaffold0272_43614*, and *scaffold0097_1805789)* and 1 auxin transport gene, *scaffold0520_237508* (**Supplementary Figure 3**). However, more genes were expressed in the radicle, plumule, and later three developmental stages of the embryonic axis, indicating the significance of auxin in specific tissues and at various stages, with most auxin-related genes exhibiting increased expression at later stages.

#### 3.3.2 Expression characteristics of ABA-related genes during seed germination

**Figure 2:**
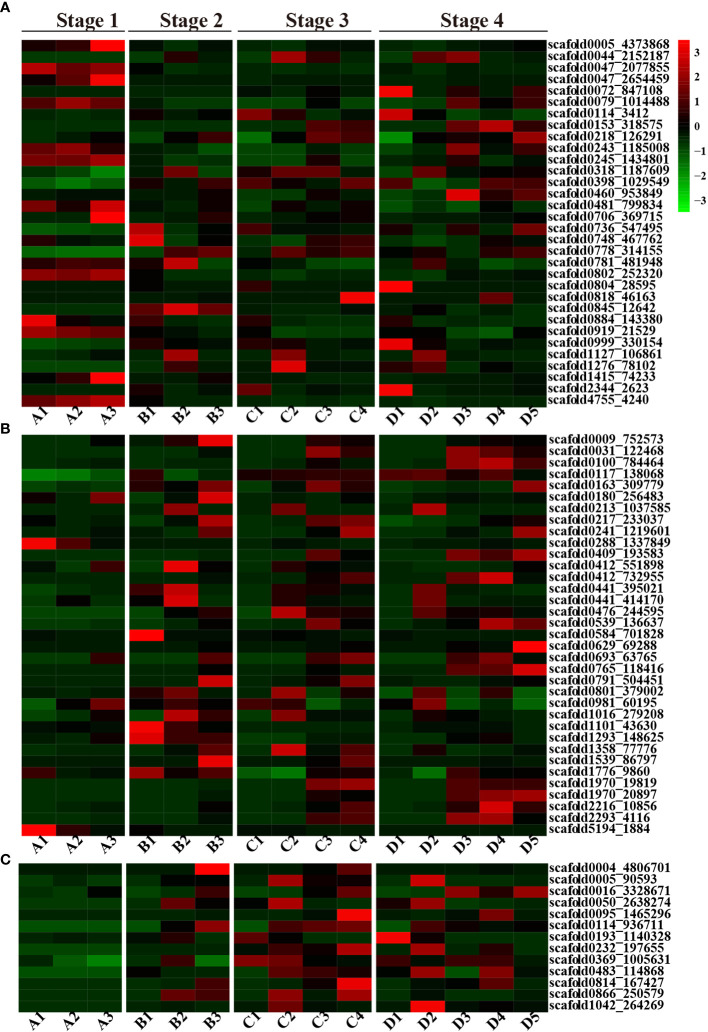
A heatmap of the ABA, GA and CTK plant hormone-related DEGs. **(A)** Heatmap of ABA-related DEGs; **(B)** Heatmap of GA-related DEGs; **(C)** Heatmap of the CTK-related DEGs; red represents upregulated genes, and green represents downregulated genes.

Overall, ABA-related genes were not actively expressed during any stage of seed germination. Some genes were exclusively expressed during the seed imbibition stage; these included three abscisic acid receptor proteins (*scaffold0005_4373868*, *scaffold0047_2077855*, and *scaffold0802_252320*) and two ABSCISIC ACID-INSENSITIVE proteins (*scaffold4755_4240* and *scaffold0047_2654459*) (**Figure 2A**). However, fewer genes exhibited limited expression in particular tissues, such as *scaffold0884_143380* (Abscisic acid receptor) in the endosperm; *scaffold1415_74233* (Cytochrome P450) and *scaffold0047_2654459* (ABSCISIC ACID-INSENSITIVE 5-like protein 2) in both the cotyledon and embryonic axes; *scaffold0706_369715* (Abscisic acid receptor PYL2) in the embryonic axis; *scaffold047_2077855* and *scaffold0079_1014488* (ABSCISIC ACID-INSENSITIVE 5-like protein 6); *scaffold0245_1434801* (Abscisic acid biosynthetic process); *scaffold0802_252320* (Abscisic acid receptor PYL8); *scaffold0919_21529* (Abscisic acid G-protein coupled receptor); *and scaffold4755_4240* (ABSCISIC ACID-INSENSITIVE 5) in the endosperm, cotyledon and embryonic axis tissues during the seed imbibition stage. However, *scaffold0005_4373868* (the abscisic acid receptor PYL9), *scaffold0047_2077855* (the abscisic acid receptor PYL8), *scaffold0802_252320* (the abscisic acid receptor), *scaffold0919_21529* (the abscisic acid G protein-coupled receptor), *scaffold1415_74233* (abscisic acid 8’-hydroxylase 4), and *scaffold4755_4240* (response to abscisic acid) were expressed only during the seed imbibition stage, and *scaffold0845_12642* (abscisic acid 8’-hydroxylase 1) was expressed only during the seed coat rupture stage (**Figure 2A**). However, *scaffold1127_106861* (cytochrome P450) and *scaffold1276_78102* (abscisic acid receptor PYL6) were expressed only in cotyledons at the later three stages.

#### 3.3.3 GA-related genes

In contrast to those of ABA-related genes, the number of GA-related genes gradually increased during seed germination and development. Fewer genes were activated during the seed imbibition stage, but a greater number of genes were highly expressed during seed coat rupture, with even more genes expressed during seedling emergence. Several GA-related genes exhibited tissue-specific expression patterns. For instance, *scaffold0629_69288*, which is annotated as gibberellin 2-beta-dioxygenase, exhibited expression in the plumule. In contrast, *scaffold0288_1337849* (gibberellin 2-beta-dioxygenase 1) and *scaffold5194_1884* (gibberellin 20-oxidase) were exclusively expressed in the endosperm during the seed imbibition stage, and *scaffold0009_752573* (gibberellin 2-beta-dioxygenase) was solely expressed in the embryonic axis at the seed coat rupture stage. Additionally, *scaffold1293_148625* (chitin-inducible gibberellin-responsive protein 1) was expressed only at the seed coat rupture stage (**Figure 2B**).

#### 3.3.4 CTK-related genes are preferentially expressed in cotyledon and embryonic axis

Interestingly, no CTK-related genes were expressed during the seed imbibition stage, but their expression increased during the subsequent three stages of seed germination. But, CTK-related genes only expressed in cotyledon and embryonic axis at seed coat rupture stage. The CTK-related genes also exhibited tissue-specific expression patterns. *Scaffold0005_90593* (the cytokinin riboside 5’-monophosphate phosphoribohydrolase LOG7), *scaffold0050_2638274* (the cytokinin riboside 5’-monophosphate phosphoribohydrolase LOG3), and *scaffold1042_264269* (the cytokinin riboside 5’-monophosphate phosphoribohydrolase LOG1) were exclusively expressed in the cotyledon at different stages, while *scaffold0004_4806701* (cytokinin dehydrogenase 1) was exclusively expressed in the embryonic axis (**Figure 2C**). In general, CTK related genes were mainly expressed in cotyledon, embryonic axis and radicle. These findings strongly suggest that CTK-related genes play a crucial role in rubber plant seed germination and development.

### 3.4 The natural rubber biosynthesis pathway activated during seed germination

**Figure 3:**
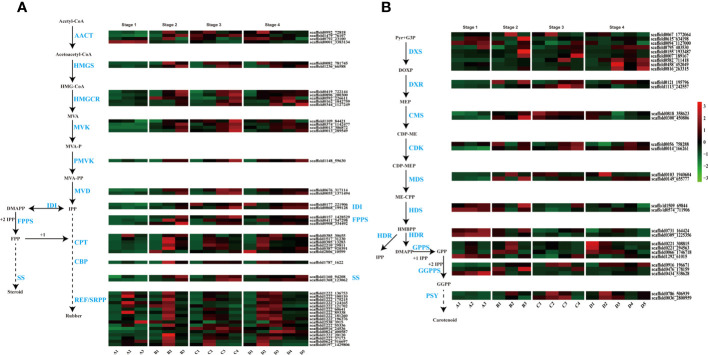
Terpenoid biosynthesis pathway and expression profiles. **(A)** MVA (mevalonate) pathway for rubber and steroid synthesis. AACT, acetyl-CoA C-acetyltransferase; HMGS, HMG-CoA synthase; HMGCR, HMG-CoA reductase; MVK, mevalonate kinase; PMVK, phosphomevalonate kinase; MVD, mevalonate pyrophosphate decarboxylase; IDI, IPP isomerase; FPPS, FPP synthase; SS, squalene synthase; CPT, cis-prenyltransferase; CBP, CPT-binding protein; REF, rubber elongation factor protein; SRPP, small rubber particle protein; **(B)** The 2-C-methyl-D-erythritol-4-phosphate (MEP) pathway for carotenoid biosynthesis. DXS, DOXP synthase; DXR, DOXP reductoisomerase; CMS, CDP-ME synthase; CDK, CDP-ME kinase; MDS, ME-CPP synthase; HDS, HMBPP synthase; HDR, HMBPP reductase; GPPS, geranyl pyrophosphate synthase; GGPPS, geranyl pyrophosphate synthase; PSY, phytoene synthase.

Natural rubber, an essential hydrocarbon polymer used in industry, primarily consists of cis-1,4-polyisoprene (Cherian et al., 2019; Yamashita and Takahashi, 2020; Calvo-Polanco et al., 2021). Two rubber biosynthesis pathways exist (Long et al., 2021). The 2-C-methyl-D-erythritol-4-phosphate (MEP) pathway supplies isopentenyl pyrophosphate (IPP) monomers for rubber synthesis, while the MVA pathway is the primary biosynthetic route for rubber and steroids in the cytosol (Chow et al., 2012). With respect to the transcriptome dataset, we identified genes associated with these two pathways in various tissues and at various seed developmental stages. A previous study indicated that latex vessels were already formed in the embryonic axis and cotyledon (Wu et al., 1990), and the expression of rubber synthesis-related genes in the cotyledon and embryonic axes was verified in this study (**Figure 3A**).

In our study, genes from these pathways exhibited differential expression, with high expression levels in the embryonic axis and cotyledon (**Figure 3**). MVA, the primary rubber synthesis pathway, exhibited increased gene activation throughout seed development, especially in the late stages of rubber biosynthesis involving *REF/SRPP* genes, where numerous genes, such as *SRPP* and *CPT*, were highly expressed in the embryonic axis and cotyledon (**Figure 3A**). *scaffold0992_72818* is an acetyl-CoA acetyltransferase (AACT) that catalyzes acetyl-CoA to produce acetoacetyl-CoA in cotyledons at later three stages, and another AACT *scaffold0001_3383134*, is expressed in only all tissues at the seed imbibition stage.

The number and expression level of genes related to latex synthesis were greater in cotyledons than in the embryonic axis. Interestingly, several *CPTs* (*scaffold0385_30655* and *scaffold0387_920391*), *CBPs* (*scaffold1160_94208* and *scaffold1368_123062*) and *REF/SRPP* (*scaffold0824_400587*, *scaffold0916_24536* and *scaffold2538_3915*) were expressed in the radicle or plumule at later two stages.

In the MEP pathway, the rate-limiting enzyme 1-deoxy-D-xylulose-5-phosphate reductoisomerase (DXR) catalyzes IPP production for rubber synthesis, and DMAPP catalyzes steroid synthesis. Two *DXR* homologs exhibited high expression during the seed imbibition stage but relatively low expression in the other three developmental stages (**Figure 3B**). These results highlight the differential expression of genes involved in rubber biosynthesis during seed germination and indicate that laticifer species are likely to be present in cotyledon and embryonic axis tissues.

### 3.5 STEM analysis of DEGs

**Figure 4:**
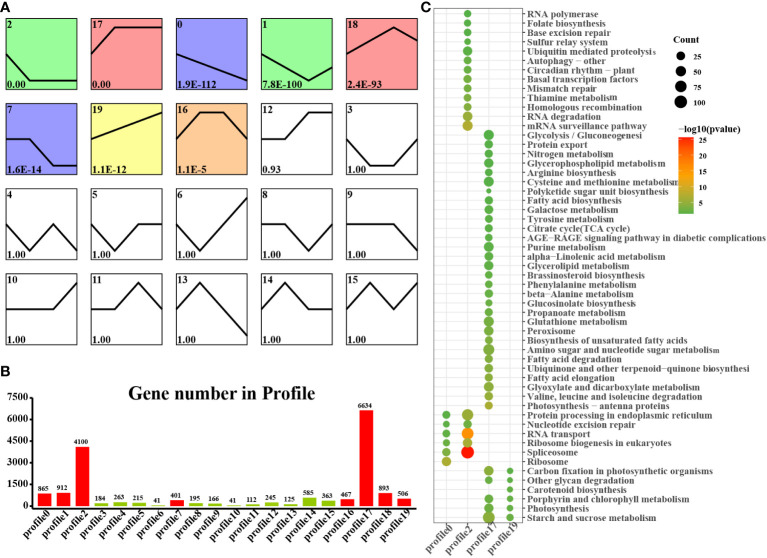
STEM analysis of cotyledon-related DEGs in all stages. **(A)** Trends in the expression profiles; **(B)** Gene numbers in the profiles; **(C)** KEGG pathway enrichment analysis of genes from the 0, 2, 17 and 19 profiles.

**Figure 5:**
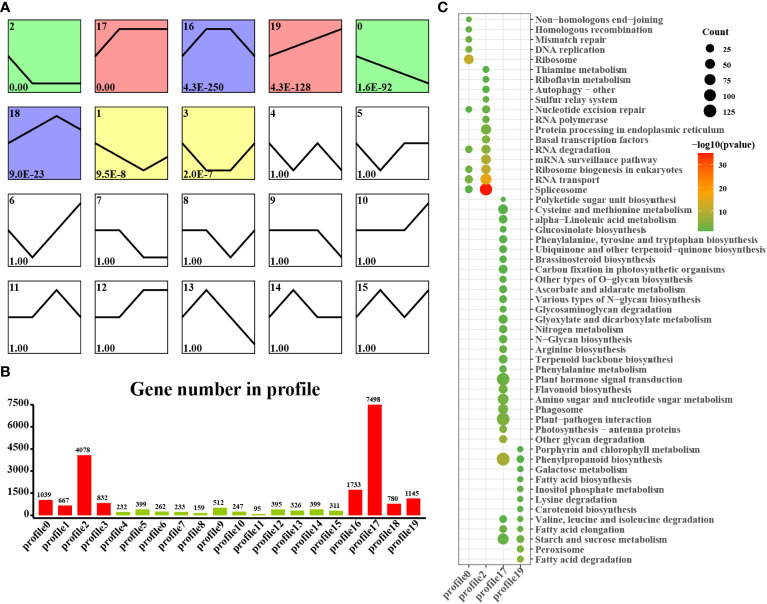
STEM analysis of embryonic axis DEGs in all stages. **(A)** Trends in the expression profiles; **(B)**, Gene numbers in the profiles; **(C)** KEGG pathway enrichment analysis of genes from the 0, 2, 17 and 19 profiles.

We found that laticifers may exist only in the cotyledon and cotyledon tissues of rubber seeds; therefore, to classify and analyze genes in cotyledon and embryonic axis tissues with similar expression trends, we utilized the short time-series expression miner (STEM) approach in this study. This analysis identified eight genes (profiles 0, 1, 2, 7, 12, 17, 18, and 19) among the significantly differentially expressed genes (DEGs) with a significance threshold of P < 0.05. Among these cotyledon profiles, Profiles 0, 2, 17, and 19 exhibited distinct and noticeable gene expression trends, warranting further investigation. In Profiles 0 and 2, gene expression showed a downward trend during seed germination, indicating downregulation during seed germination. Conversely, the expression of profiles 17 and 19 demonstrated an overall upward trend, indicating upregulation during seed germination. Profile 17 revealed the activation of pathways related to nutrient supply and recycling, including the TCA cycle, endocytosis, and nitrogen metabolism in the cotyledon. Moreover, Profile 19 indicated the activation of energy metabolism pathways, such as oxidative phosphorylation, photosynthesis, and carbon fixation pathways, in the cotyledons (**Figure 4**). Similarly, on the embryonic axis, the expression of the genes in profiles 0 and 2 tended to decrease, similar to what was observed in the cotyledons. In contrast, profiles 17 and 19 in the embryonic axis were associated with the enrichment of pathways related to terpenoid biosynthesis, plant hormone signal transduction, phenylpropanoid biosynthesis, flavonoid biosynthesis, and the phagosome. These findings suggest the activation of secondary metabolism pathways, including the MVA and MEP pathways, in the embryonic axis during seed germination (**Figure 5**).

### 3.6 Transcription factors involved in the cotyledon and embryonic axis

**Figure 6:**
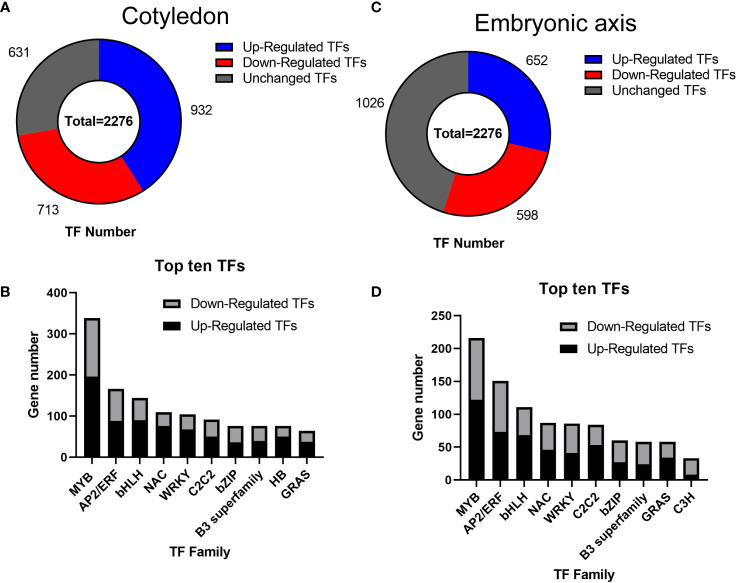
Transcription factor analysis. **(A)** Number of transcription factors in the cotyledon; **(B)** Number of genes associated with the top ten transcription factors in the cotyledon; **(C)** Number of transcription factors in the embryonic axis; **(D)** Number of genes associated with the top ten transcription factors on the embryonic axis. MYB, myeloblastosis; AP2/ERF, APETALA 2/ethylene-responsive element binding factor; bHLH, basic helix-loop-helix; NAC, NAM-ATAF1/2-CUC1/2; WRKY, WRKY domain protein; C2C2, C2C2 zinc finger; bZIP, basic-region leucine zipper; B3 superfamily, B3 domain transcription factor; HB, homeobox; GRAS, Gibberellin insensitive-RGA25-SCR26 family; C3H, CCCH zinc finger protein.

**Figure 7:**
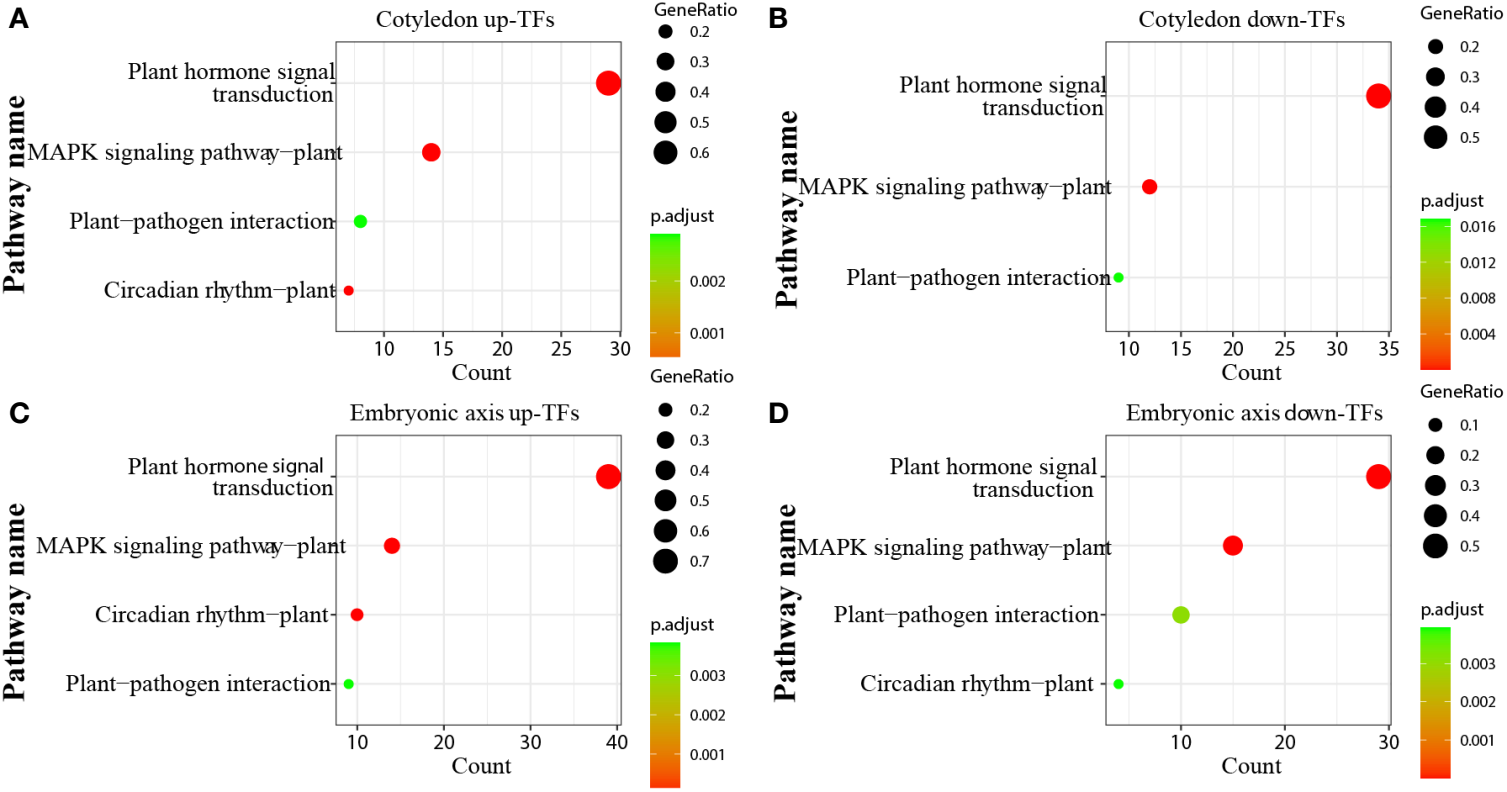
KEGG enrichment analysis of transcription factors. **(A)** KEGG enrichment analysis of up-TFs in cotyledons; **(B)** KEGG enrichment analysis of downregulated TFs in cotyledons; **(C)** KEGG enrichment analysis of up-TFs in the embryonic axis; **(D)** KEGG enrichment analysis of downregulated TFs in the embryonic axis.

Transcription factors (TFs) play significant roles in regulating tissue development, natural rubber biosynthesis, and laticifer differentiation (Iglesias-Fernandez et al., 2013; Lee et al., 2015; Wang et al., 2017; Yu et al., 2017; Kumpeangkeaw et al., 2019). To analyze the possible relationships between transcription factors and laticifer differentiation in cotyledons and cotyledons, according to the Plant Transcription Factor Database (PlantTFDB), a total of 2,276 TFs were predicted for the rubber tree. Among these genes, 713 were upregulated, and 932 were downregulated at all stages of cotyledon development (**Figure 6A**). The top 10 transcription factor families in the cotyledons included the MYB, AP2/ERF, bHLH, NAC, WRKY, C2C2, bZIP, B3 superfamily, HB, and GRAS (**Figure 6B**). In all stages of embryonic axis development, 589 TFs were upregulated, and 652 were downregulated (**Figure 6C**). The top 10 transcription factor families observed in the embryonic axis were similar to those in the cotyledon but with varying numbers (**Figure 6D**). Furthermore, pathways containing both up- and downregulated TFs exhibited enrichment in processes related to plant hormone signal transduction, MAPK signaling, plant−pathogen interactions, and circadian rhythm (**Figure 7**). According to the laticifer-specific expression data (unpublished), *scaffold0675_129662* (auxin response factor), *scaffold0778_314155* (ABSCISIC ACID-INSENSITIVE), *scaffold0941_312542* (DELLA protein, GA pathway), *scaffold1421_86909* and *scaffold0017_1455021* (AP2) and *scaffold0332_497007*, *scaffold0148_1097530*, and *scaffold1297_134611* (MYB) were upregulated in the cotyledons. In addition, *scaffold0675_129662*, *scaffold1230_90996* (auxin response factor), *scaffold0778_314155* (ABSCISIC ACID-INSENSITIVE), *scaffold0941_312542* (DELLA protein, GA pathway), *scaffold1421_86909*, *scaffold0017_1455021*, *scaffold0037_175787*, *scaffold0013_532383* (AP2), *scaffold0332_497007*, *scaffold0148_1097530*, and *scaffold0073_653695* (MYB) were upregulated in the cotyledons. These TFs, which are specifically expressed in laticifer, may regulate laticifer differentiation and rubber synthesis.

### 3.7 Laticiffer cells staining

**Figure 8:**
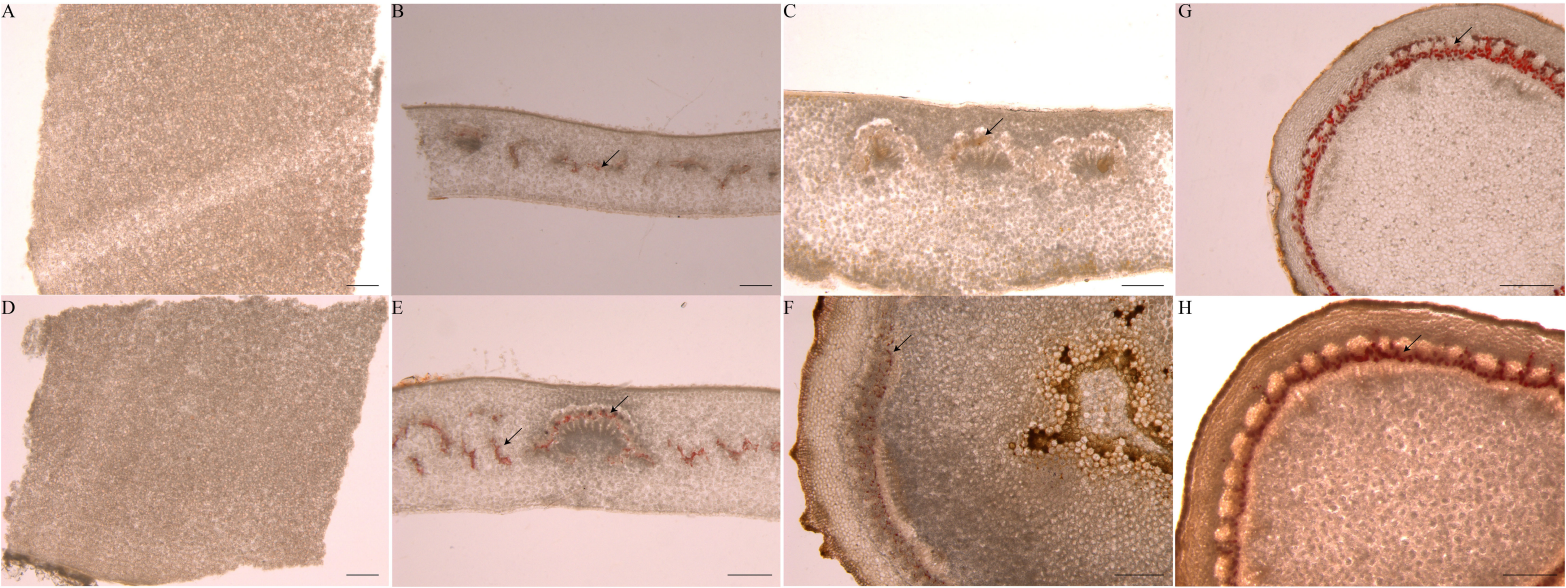
Identification of laticifer cells in different rubber seed tissues by. The laticifer cells (black arrowheads) were colored in red. **(A–C)** staining of the endosperm, cotyledon and embryonic axis respectively before germination; **(D–H)** staining of the endosperm, cotyledon, embryonic axis, radicle and plumule respectively after germination. Scale bars = 1 mm.

There are few reports about laticifer cells during seed germination. We identified laticifer cells of the endosperm, cotyledon and embryonic axis before germination and the endosperm, cotyledon, embryonic axis, radicle, and plumule after germination by staining with Oil Red O. The result showed that laticifer cells were observed in the cotyledon and embryonic axis but not in endosperm before seed germination (**Figures 8A–C**). Laticifer cells were observed in all tissues except endosperm after seed germination (**Figures 8D–H**). The results reveled that the original laticifer cells are likely to develop with seed formation.

### 3.8 Validation of DEGs by qPCR

**Figure 9:**
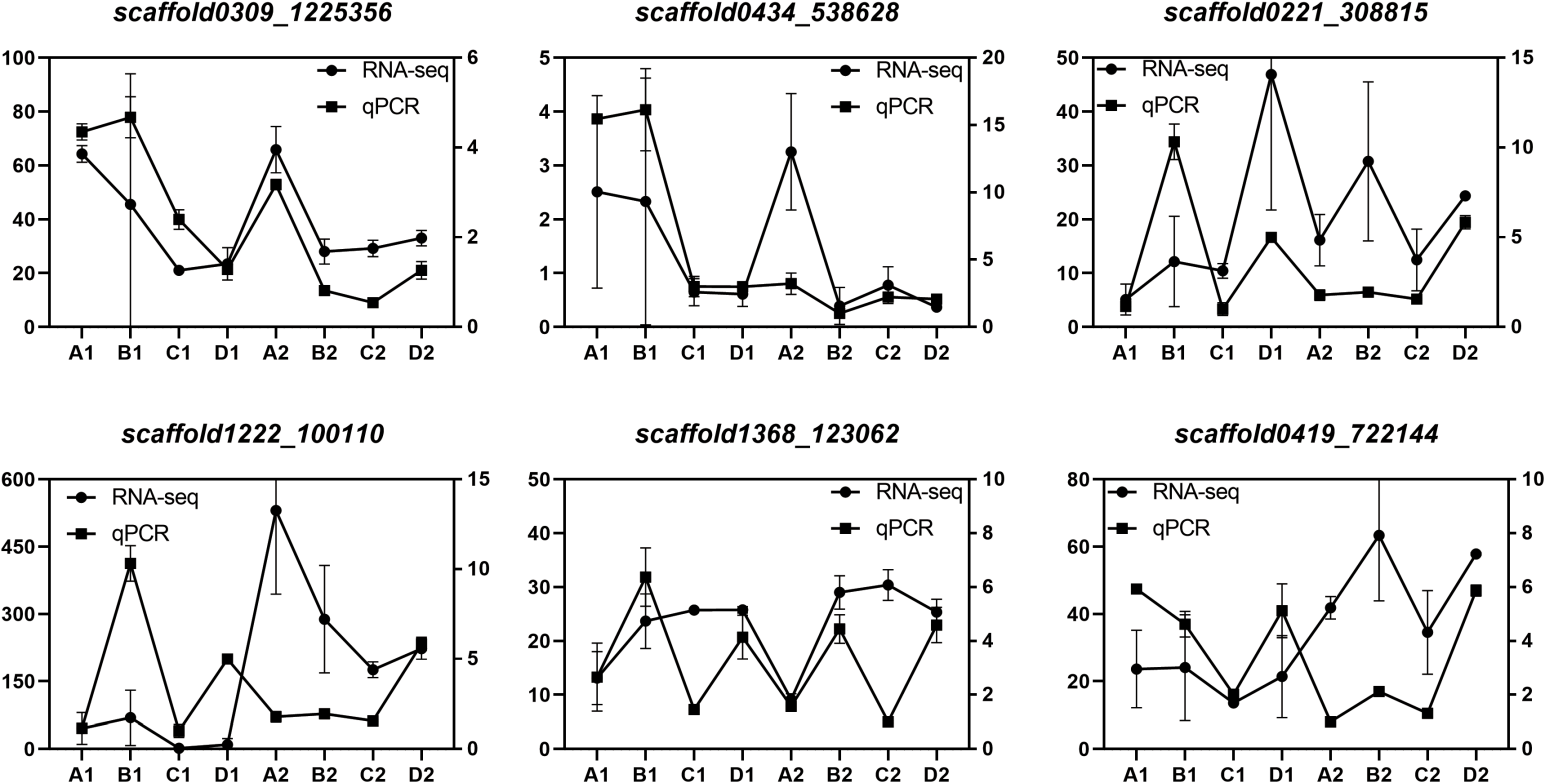
Verification of DEGs by qPCR Expression levels of 6 genes. The relative expression levels of each gene were measured using the 2^–ΔΔCT^ method. *Scaffold0221_308815*, *GPPS*; *scaffold0434_538628*, *GGPPS*; *scaffold0309_1225356*, *HDR*; *Scaffold1222_100110*, *REF/SRPP*; *scaffold1368_123062*, *SS*; *scaffold0419_722144*, *HMGCR*.

We validated the expression patterns of six DEGs identified through RNA-Seq analysis in two tissues (endosperm and cotyledon) and four seed developmental stages (A, B, C, and D). These six genes, namely, *HDR* (*scaffold0309_1225356*), *GGPPS* (*scaffold0434_538628*), *GPPS* (*scaffold0221_308815*), *REF/SRPP* (*scaffold1222_100110*) are involved in the MEP pathway, and *SS* (*scaffold1368_123062*), *HMGCR* (*scaffold0419_722144*) are involved in the MVA pathway. The qPCR analysis results closely matched the transcriptome data (**Figure 9**), confirming the reliability of the DEG identification results.

## 4 Discussion

Scientists have focused on latex production traits in rubber trees, the main natural rubber-producing plants. It remains unclear whether latex production initiation and laticifer differentiation are related to seed germination. Our study analyzed DEGs during seed germination and revealed that rubber synthesis and seed germination commence simultaneously.

### 4.1 Phytohormone signaling pathways and laticifer differentiation

Study reported that hormone-related genes promoted the differentiation of secondary laticifer cells (Loh et al., 2019). IAA was shown to promote laticifer differentiation (Suri and Ramawat, 1995). In our study, we observed enrichment of phytohormone pathways during seed germination, with a particular emphasis on the auxin signaling pathway, which exhibited significant changes. Only seven genes, including six auxin-responsive genes and one auxin transporter gene (*scaffold0520_237508*), were consistently expressed across all tissues during the four seed germination stages. The auxin transporter gene is likely involved in the development of laticifers and other tissues. Previous research has shown that *ARF5* and *WOX13*, which are associated with the auxin signaling pathway, are highly expressed in newly developed stem tissues of high latex–yield rubber tree clones, suggesting that increased expression of *ARF5* and *WOX13* may promote laticifer differentiation (Sae-Lim et al., 2019). Most of these genes were expressed in the embryonic axis, where laticifer cells exist. The expression of the *scaffold0675_129662* and *scaffold1230_90996* TFs (involved in the auxin pathway), which are specifically expressed in laticifer, increased in both the cotyledon and embryonic axes, suggesting that auxin likely plays a role in laticifer differentiation or rubber synthesis.

As a stress-related hormone, the ABA signaling pathway mediates laticifer differentiation in rubber trees (Tian et al., 2015). In the present study, ABA-related genes exhibited specific expression patterns in different tissues and at different stages. For example, *scaffold0050_2638274* (the PYL6 ABA receptor) was exclusively expressed in the endosperm, while *scaffold0047_2077855* (an ABA receptor), *scaffold0245_1434801* (associated with abscisic acid biosynthesis), *scaffold4755_4240* (ABSCISIC ACID-INSENSITIVE 5), and *scaffold0919_21529* (the abscisic acid G protein-coupled receptor) were expressed during the seed coat rupture stage. G protein-coupled receptor (GPCR) is a crucial developmental regulator that acts as a regulator of G protein signaling and participates in various developmental processes, including seed germination (Kansup et al., 2013). We observed increased expression of ABA-related genes in the endosperm during the seedling emergence stage, suggesting their vital role in nutrient supply during late seed germination (the seedling emergence stage). Additionally, several ABA-related genes were expressed in the embryonic axis during the seed imbibition stage, and *scaffold0778_314155*, a TF involved in the ABA pathway that is specifically expressed in laticifer, was upregulated in the cotyledon and embryonic axes, suggesting that ABA likely plays a role in laticifer differentiation or rubber synthesis.

GA-related genes exhibited low expression during the seed imbibition stage, with two genes, *scaffold0288_1337849* (gibberellin 2-beta-dioxygenase 1) and *scaffold5194_1884* (gibberellin 20-oxidase), exclusively expressed in the endosperm. GA is known to promote starch catabolism and reduce the accumulation of stored proteins (Hu et al., 2021). These two genes likely promote gibberellin synthesis and accumulation. *Scaffold0629_69288* (gibberellin 2-beta-dioxygenase) was expressed only in the plumule, suggesting its role in plumule development. GA-related genes were predominantly expressed during the seed coat rupture stage in three tissues: endosperm, cotyledon, and embryonic axis. *scaffold0941_312542*, a TF involved in the GA pathway that is specifically expressed in the laticifer, was upregulated in the cotyledon and embryonic axes, indicating that GA may regulate laticifer differentiation or rubber synthesis.

The differentiation of laticifers depends on cytokinin levels (Suri and Ramawat, 1995). However, the expression pattern of CTK differed from that of the aforementioned hormones. CTK-related genes were not expressed during the seed imbibition stage, indicating that CTK likely does not function in the early stages of rubber tree seed germination. While CTK is abundant in the endosperm of rice and maize during seed germination (Rijavec and Dermastia, 2010), CTK-related genes are scarcely activated in the endosperm of rubber plants at all seed germination stages. This finding suggested that CTK levels are likely low. CTK-related genes were highly expressed in the radicle and cotyledon (**Figure 5C**), suggesting that CTK may promote the development of the radicle and cotyledon during seed germination. Furthermore, *in vitro* laticifer differentiation in Calotropis procera is dependent on cytokinin (Suri and Ramawat, 1995), and high expression of CTK-related genes likely promotes laticifer differentiation in cotyledons.

Studies have shown that brassinosteroids (BRs) pathway is related to plant morphogenesis (Wang et al., 2012). In this study, we analyzed the gene expression changes of BRs pathway during seed germination, but only three genes were identified in DEGs. And just two genes, scaffold0123_1858764 and scaffold0441_941076, differentially expressed in cotyledon at early period of seed germination. It is suggested that BRs may not be the main hormone in the differentiation of early laticifer cells (**Supplementary Figure 4**).

The response to different hormones during seed germination may link hormones to seed germination rate and laticifer development, and provide farmers with convenient breeding methods. Meanwhile, the analysis of hormone related genes expression may provide effective clues for us to promote the differentiation of laticifer cells in seeds by certain hormones in the future.

### 4.2 Rubber biosynthesis and laticifer differentiation during seed germination

According to the structure, laticifers in rubber tree is divided into articulated and non-articulated laticifers (Sando et al., 2009). Non-articulated laticifers derive from single cell, and articulated laticifers formed by the fusion of multiple cells (Castelblanque et al., 2016).

Exogenous PSK promoted the differentiation of laticifers in the epicormic shoots of rubber tree(Chao et al., 2023). While there are limited reports on the relationship between seed germination and rubber synthesis, our study revealed differential expression of genes related to rubber biosynthesis in specific tissues and at specific seed germination stages. Previous studies suggested that laticifer initiation occurs in tissues such as cotyledons and on the embryonic axis (Bruni, A et al., 1978). Similarly, laticifer initiation in the early stages of laticifer cell differentiation was found in the cotyledon and embryonic axes of rubber plants (Castelblanque et al., 2016), and our laticifer cells staining confirms that laticifer cells were already present in the cotyledon and embryonic axes of seeds before germination (**Figures 8B, C**). We hypothesized that the initiation of differentiation of laticifer cells began with seed germination. Laticifer initiation involves invasive growth between adjacent cells and subsequent branching and elongation until mature laticifer formation occurs (Tan et al., 2019; Johnson et al., 2021). In our study, during seed germination, *SRPP*, *CBP*, *CPT*, and genes related to rubber synthesis were preferentially expressed in the cotyledon and embryonic axes and subsequently expressed in the radicle, while little was expressed in the radicle, indicating that laticifer differentiation occurred first in the cotyledon and embryonic axes, followed by the radicle and plumule. Moreover, more CPT and REF/SRPP genes were expressed in the cotyledons than in the other cotyledons at all stages. Moreover, *REF/SRPP* was primarily expressed in cotyledons during the first three stages and then significantly elevated in expression in the fourth stage, indicating that cotyledons may contain more laticifers than embryos at the early stage. However, *CPTs* (*scaffold0385_30655* and *scaffold0387_920391*), *CBPs* (*scaffold1160_94208* and *scaffold1368_123062*), and *REF/SRPP* (*scaffold0824_400587*, *scaffold0916_24536*, and *scaffold2538_3915*) were expressed in the radicle or plumule at the radicle protrusion or seedling emergence stages, indicating that these genes may play a role in the differentiation of different tissues in laticifers. Previous studies did not detect latex synthesis during seed germination, but in the present study, we showed that *CPT* and *REF/SRPP*, which are specifically expressed in laticifers, were expressed at the transcriptional level in the cotyledon and embryonic axes during seed germination. These results verified that laticifers are likely present in the seed. In the MEP pathway, HDR can catalyze the transformation of HMBPP to IPP, which is the substrate of rubber synthesis, but *HDR* has a very low expression level in the latter three stages, which is contrary to the expression levels of a large number of rubber synthesis-related genes in the MVA pathway. These findings indicate that the IPP produced by the MEP pathway is not amenable to rubber synthesis. Study reported that the laticifer density can be used as a screening marker for high-yield rubber tree breeding and may be shorten the rubber tree breeding period(Tan et al., 2019). Therefore, the number of original laticifer cells may determine its production after maturity. In this study, we found that IAA, GA and CTK related genes expressed highly in laticifer cells containing tissues. It is possible that these hormones are involved in the initial development of laticifer cells. Hormone-treated seeds may be a new breeding method to enhance the initial development of laticifer cells and thus enhance their latex-producing potential in mature rubber tree.

### 4.3 Rubber biosynthesis and laticifer differentiation during seed germination: specific transcription factors are expressed in laticifer cells

A variety of transcription factors have been identified in the cotyledon and embryonic axes. As important regulators, TFs play crucial roles in cell differentiation and embryogenesis (Long et al., 2006). In this study, TFs associated with the cotyledon and embryonic axes were enriched, with the three genes being MYB, AP2/ERF, and bHLH (**Figure 6**). Previous studies have shown that the AP2/ERF family is involved in cell differentiation and dedifferentiation (Iwase et al., 2011; Marsch-Martinez et al., 2006), while MYB and bHLH are involved in cell differentiation (Gómez et al., 2009; Katayama et al., 2015; Tominaga et al., 2014). Screening for the specific expression of these transcription factors in laticifer cells may be related to laticifer differentiation or rubber synthesis. We screened several laticifer-specifically expressed TFs, including those upregulated both in cotyledons and in the embryonic axis. As MYB-type transcription factors, *scaffold0332_497007* and *scaffold0148_1097530* are both upregulated in the laticifer cotyledon and embryonic axes. Similarly, *scaffold0675_129662* (auin response factor), *scaffold0778_314155* (ABSCISIC ACID-INSENSITIVE) and *scaffold0941_312542* (GA pathway) were upregulated in the laticifer cotyledon and embryonic axes. The above genes, which are specifically expressed in laticifer, are likely involved in laticifer differentiation or rubber biosynthesis.

## 5 Conclusions

In conclusion, our findings provide valuable insights into natural rubber synthesis and laticifer differentiation during seed germination. Genes related to rubber biosynthesis exhibited tissue-specific expression patterns during seed germination. The MYB-type TFs *scaffold0332_497007* and *scaffold0148_1097530* and the plant hormone-related TFs *scaffold0675_129662* (Auxin response factor), *scaffold0778_314155* (ABSCISIC ACID-INSENSITIVE) and *scaffold0941_312542* (GA pathway) are likely to play vital roles in laticifer differentiation or rubber biosynthesis.

## Data availability statement

The raw data is available on NCBI, and the accession number is PRJNA1060902.

## Author contributions

BH: Writing – original draft, Writing – review & editing. NY: Investigation, Writing – review & editing. ZZ: Data curation, Writing – review & editing. XS: Investigation, Writing – review & editing. YQ: Methodology, Writing – review & editing. YF: Data curation, Writing – review & editing. XL: Data curation, Writing – review & editing.

## Supplementary material

Supplementary Figure 1 Rubber seed samples for RNA-seq and identification of novel transcripts. **(A)** Samples from four seed germination stages are stage 1, seed imbibition; stage 2, seed coat rupture; stage 3, radicle protrusion; and stage 4, seedling emergence (represented by **(A–D)**, respectively); **(B)** Five tissues, endosperm, cotyledon, embryonic axis, radicle and plumule (represented by 1, 2, 3, 4 and 5, respectively), were collected during seed germination processing, and three biological replicates were performed. Bars are 1 cm.

Supplementary Figure 2 UpSet diagram analysis of DEGs associated with the endosperm, cotyledon and embryonic axes. log2(fold change) >1, p<0.05.

Supplementary Figure 3 The heatmap of auxin-related DEGs. Red represents upregulated genes, and green represents downregulated genes.

Supplementary Figure 4 The heatmap of BRs-related DEGs. Red represents upregulated genes, and green represents downregulated genes.
