# Supplementary figures and images for: Transcriptome analysis reveals the molecular mechanisms of rubber biosynthesis and laticifer differentiation during rubber seed germination
Source: Front Plant Sci. 2024 Jan 24;15:1337451. doi: 10.3389/fpls.2024.1337451 (PMC10847244; doi:10.3389/fpls.2024.1337451)

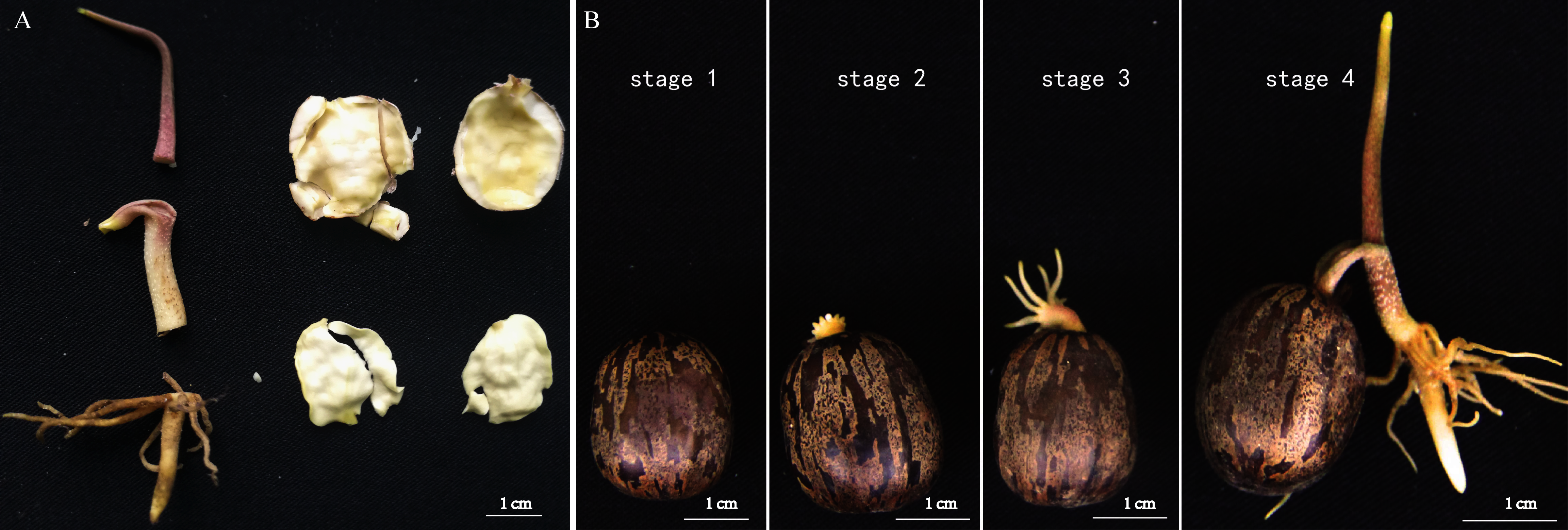

Supplement: Supplementary Figure 1 — Rubber seed samples for RNA-seq and identification of novel transcripts. (A) Samples from four seed germination stages are stage 1, seed imbibition; stage 2, seed coat rupture; stage 3, radicle protrusion; and stage 4, seedling emergence (represented by (A–D), respectively); (B) Five tissues, endosperm, cotyledon, embryonic axis, radicle and plumule (represented by 1, 2, 3, 4 and 5, respectively), were collected during seed germination processing, and three biological replicates were performed. Bars are 1 cm. [file Image_1.tif]

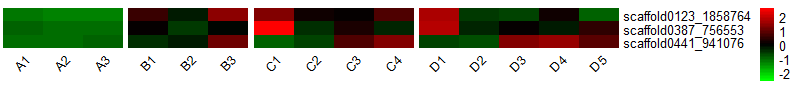

Supplement: Supplementary Figure 2 — UpSet diagram analysis of DEGs associated with the endosperm, cotyledon and embryonic axes. log2(fold change) >1, p<0.05. [file Image_2.tif]

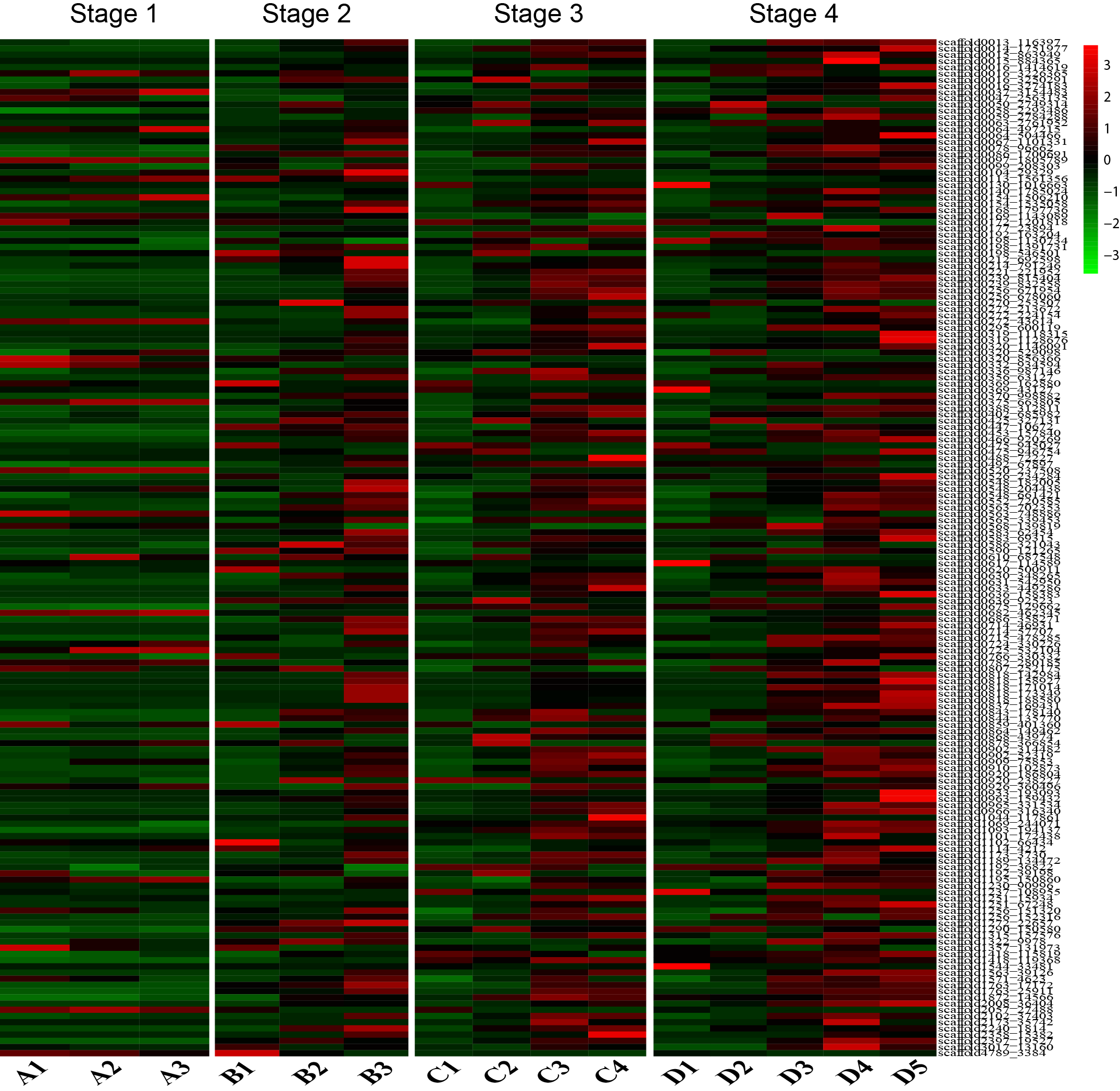

Supplement: Supplementary Figure 3 — The heatmap of auxin-related DEGs. Red represents upregulated genes, and green represents downregulated genes. [file Image_3.tif]
